# HIV epidemic in Far-Western Nepal: effect of seasonal labor migration to India

**DOI:** 10.1186/1471-2458-11-310

**Published:** 2011-05-13

**Authors:** Naveen K Vaidya, Jianhong Wu

**Affiliations:** 1 Department of Applied Mathematics, University of Western Ontario, London, ON, Canada; 2 Theoretical Biology and Biophysics Group, Los Alamos National Laboratory, Los Alamos, NM, USA; 3 Center for Disease Modeling, Department of Mathematics and Statistics, York University, Toronto, ON, Canada

## Abstract

**Background:**

Because of limited work opportunities in Nepal and the open-border provision between Nepal and India, a seasonal labor migration of males from Far-Western Nepal to India is common. Unsafe sexual activities of these migrants in India, such as frequent visits to brothels, lead to a high HIV prevalence among them and to a potential transmission upon their return home to Nepal. The present study aims to evaluate the role of such seasonal labor-migration to India on HIV transmission in Far-Western Nepal and to assess prevention programs.

**Methods:**

An HIV epidemic model was developed for a population in Far-Western Nepal. The model was fitted to the data to estimate the back and forth mobility rates of labor-migrants to India, the HIV prevalence among migrants and the HIV transmission rate in Far-Western Nepal. HIV prevalence, new infections, disease deaths and HIV infections recruited from India were calculated. Prevention programs targeting the general population and the migrants were evaluated.

**Results:**

Without any intervention programs, Far-Western Nepal will have about 7,000 HIV infected individuals returning from India by 2015, and 12,000 labor-migrants living with HIV in India. An increase of condom use among the general population from 39% to 80% will reduce new HIV infections due to sexual activity in Far-Western Nepal from 239 to 77. However, such a program loses its effectiveness due to the recruitment of HIV infections via returning migrants from India. The reduction of prevalence among migrants from 2.2% to 1.1% can bring general prevalence down to 0.4% with only 3,500 recruitments of HIV infections from India.

**Conclusion:**

Recruitment of HIV infections from India via seasonal labor-migrants is the key factor contributing to the HIV epidemic in Far-Western Nepal. Prevention programs focused on the general population are ineffective. Our finding highlights the urgency of developing prevention programs which reduce the prevalence of HIV among migrants for a successful control of the HIV epidemic in Far-Western Nepal.

## Background

Since the first case in July 1988 [1-3], the reported number of HIV infections in Nepal has gradually increased to 10,546 HIV cases and 1,610 AIDS cases as of December 2007 [4]. Until the late 1990s, Nepal was classified as having a low-level epidemic. However, after 1997, Nepal has been experiencing a concentrated epidemic with rapid spread amongst high-risk groups [2-4]. Even though the HIV prevalence among the general population has been estimated to be low (~0.5%) [4,5], among the high-risk groups it varies from 1-3% among female sex workers (FSWs) in Kathmandu (Capital of Nepal), 2.8% among migrants returning from Mumbai, India, 3.3% among urban-based males having sex with males (MSMs) and 10% among male migrants returning to Doti (a Far-Western district of Nepal) to 34.7-58% among injection drug users (IDUs) in Kathmandu [2,4,6-9]. Among these HIV risk factors, a high rate of seasonal migration of the male population to India has recently been extremely threatening in the Far-Western districts of Nepal such as Doti, Achham, Dang, Kanchanpur and Kailali [4,7-9]. Nepal's 2007 United Nations General Assembly Special Session (UNGASS) report indicates that labor migrants make up 41% of the total known HIV infections in the country [4].

Because of limited work opportunities in Nepal and the open-border provision between Nepal and India, 1.5 to 2 million Nepalis have been estimated to migrate to India for seasonal and long-term work. These especially include labor migrants from Far-Western Nepal [4,8-10]. 50-80% of households in some communities of this region have at least one family member working in India, and most of these migrants seasonally return home [11,12]. Their major destinations are Mumbai, Panjab and Chenai in India, where HIV prevalence among sex workers is high (for example, there was 71% prevalence among the sex workers of Mumbai in 1997) [7,9,13]. Their unsafe sexual activities in India - for example, frequent brothel visits, mostly after drinking alcohol [7,8] - make for a high HIV prevalence among these migrants group. Upon their return home, in addition to transmitting HIV to their wives, they also may transmit to other women because their new social status affords them the chance to have extramarital sex, for example during frequently occurring local festivals and *Deuda *(a popular local cultural event) [7,8,12].

While there are many existing mathematical models developed to understand the dynamics of HIV/AIDS in different parts of the world [14-19] and there are some field surveys [7,8,20,21], no attempt has been made to mathematically study the epidemic of HIV/AIDS in Far-Western Nepal. In this study, we developed a mathematical model to evaluate effects of seasonal labor migrants to India on the spread of HIV/AIDS in Far-Western Nepal. We estimated key parameters by fitting our model to the estimated data. We also assessed two prevention programs, one targeted at the general population and another at the migrants.

## Methods

### HIV Data

It is very difficult to gather data sources in the Far-Western region of Nepal, partly because the Far-Western region, on top of being economically the least developed zone of the country, was the hot spot for the battle during the period of Maoist insurgency (1996-2006). Based on publicly available data of HIV prevalence of the whole country [5,22] and the literature survey [5,7,8,11,22-26], we estimated yearly time-series of the HIV prevalence in Far-Western Nepal. For the years from 1990 to 2007, the total HIV infected adult population of the whole country can be obtained from the estimates in [5,22]. The Far-Western region, which is one of the four epidemic regions of the country, constitutes 16% of the total HIV infections [23]. Moreover, the demographic data from the three 10-year population census indicate 8.8% (Pop-1981), 9.1% (Pop-1991) and 9.5% (Pop-2001) of the country's population belonging to Far-Western Nepal [26]. Using these data, we estimated HIV-prevalence in Far-Western Nepal from 1990 to 2007 (Table 1).

**Table 1:** Estimated adult (15-49 age group) HIV prevalence % in Far-Western Nepal during the year from 1990 to 2007.

**Year:**	1990	1991	1992	1993	1994	1995	1996	1997	1998	1999	2000	2001
**Prevalence %:**	0.05	0.10	0.15	0.22	0.32	0.40	0.53	0.61	0.67	0.72	0.76	0.79
**Year:**	2002	2003	2004	2005	2006	2007						
**Prevalence %:**	0.81	0.81	0.83	0.83	0.83	0.83						

### SIM Model

**Figure 1:**
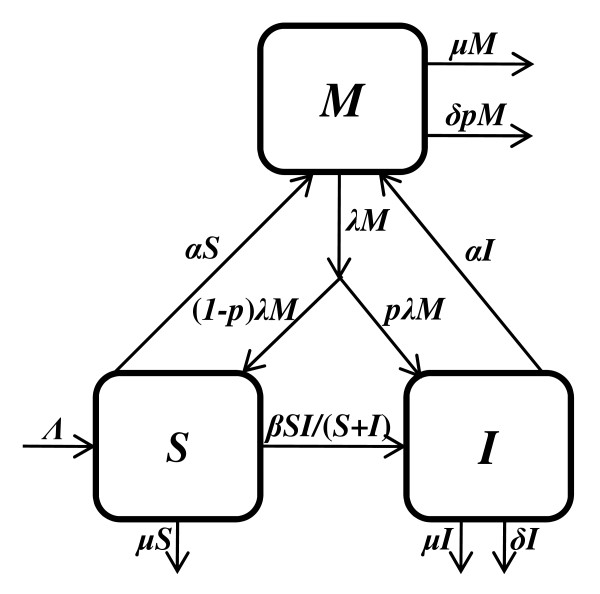
**A schematic diagram of SIM model**. The boxes represent cohorts of individuals and the arrows represent disease transmission, maturation, migration or death. *S*, *I *and *M *represent susceptible, infected with HIV and migrant workers in India, respectively.

A schematic diagram of our SIM model is shown in Figure 1. The population of interest, i.e. sexually active population of age 15-49, is subdivided into three mutually exclusive and collectively exhaustive compartments: susceptible to HIV (*S*), infected with HIV (*I*) and migrant workers in India (*M*). Since our main objective is to observe the effects of seasonal labor migration on HIV epidemic and also due to lack of sufficient data, we only consider the disease transmission and omit the disease progression (i.e. only one stage of the HIV). In Far-Western Nepal, the dominated means of HIV transmission is heterosexual contact.

The parameters *α *and *λ *are the rates of migration to India and from India, respectively. Returned migrants enter into the HIV infected and susceptible compartments with rates *p *and *1-p*, respectively. Here, *p *denotes the portion of HIV positive individuals among migrants in India. *μ *and *δ *denote the natural death rate and the disease death rate, respectively. We take *Λ *as a rate of new entries into the population of interest, i.e., the maturation rate for sexual activity. We model disease transmission and population changes over time using a system of nonlinear differential equations as follows:

The change over time in the size of the susceptible population in Far-Western Nepal is given by:(1)

Here, the HIV transmission rate, *β*, is a function of the average number of sexual partners, *n*, the average condom use rate, *c*, and infectivity (i.e., the probability per unprotected sexual partnership that an infected individual transmits the disease to a susceptible individual), *θ*, according to formula: *β = n(1-c)θ *[17].

The change over time in the number of HIV infected population in Far-western Nepal is given by:(2)

The change over time in the number of migrants from Far-western Nepal working in India is given by:(3)

Note that our model can further be extended by dividing the total migrant group, *M*, into susceptible and infected groups. However, among the male migrants considered in this study, a new HIV infection in India is not due to sexual contacts between susceptible and infected migrants, but is due to unsafe sexual activities such as frequent brothel visits [7,8]. Therefore, the model having susceptible and infected migrant groups separately will require simply a linear term, rather than non-linear term, representing a flow with a constant rate from the susceptible migrant group to the infected migrant group. In this case, considering a single parameter, as *p *in our model, to represent the HIV prevalence among migrants can produce equivalent results. Moreover, a representation of HIV prevalence among migrants as a single parameter makes it easier to compare our estimates with the field survey studies.

Taking *X *= *S *+ *I *= *N - M*, the total population living back home in Far-Western Nepal, the model can equivalently be expressed as(4)(5)(6)

See the Appendix for the expression of HIV prevalence in a long-term and further model analysis.

Even though Nepal is facing the challenge of controlling HIV among its high-risk groups, the disease prevalence among the general population remains low (less than 0.5% in 2007 [4,5]). Therefore, the model simplified to a low prevalence scenario can reasonably approximate the dynamics of HIV/AIDS in Far-Western Nepal. In the low prevalence setting, we can solve the model to obtain close form solutions *S*, *I *and *M *(See Appendix).

### Parameter Estimation

**Figure 2:**
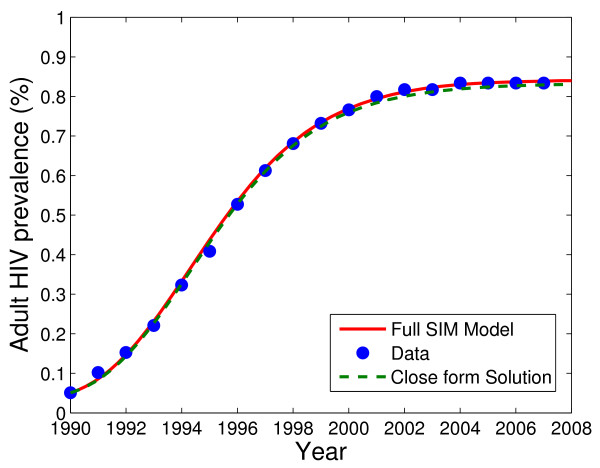
**Dynamics of adult HIV-prevalence (%) in Far-Western Nepal predicted by the model in low-prevalence setting [solid red curve] and the full SIM model [dashed green curve] along with the data [blue filled circle]**.

Far-Western Nepal, one of the five development regions (Eastern, Central, Western, Mid-Western and Far-Western) of the country, has a population of 2,191,330 according to the 2001 census [26,27]. In 1990 [28], the total population of Far-Western Nepal was 1,679,301 [28], among which ~55.0% belong to the 15-64 age group [29]. Assuming uniform distribution of the population over age-groups, we obtained ~38% in 15-49 age group, which gives *N *(0) = 638,130. The initial time *t *= 0 represents the year 1990. Studies show that 50-80% (65% on average) of households in some Far-Western communities have at least one family member working in India [11,12]. According to a survey [30], a majority of the households were found to have less than 6 members. Taking an average of 5 members in each household we obtain 335,860 families in the Far-Western region, 65% of which have one family member working in India (i.e. 218,310 persons). Therefore, we take *M *(0) = 218,310, giving *S *(0) + *I *(0) = 419,820. With the prevalence 0.05% in 1990 (Table 1, Figure 2), we obtained *I *(0) = 210 and *S *(0) = 419,610.

Nepal's life expectancy at birth is 60.56 years [31]. With 15-year as the age of entering into the sexually active population, a remaining life-time of the healthy population following recruitment to the sexually active group is 60.56-15 = 45.56 years. This gives 1/*μ *= 45.56 years, i.e. the natural death rate is *μ *= 0.022 y^-1^. Realizing that the life-expectancy at age 15 is usually higher than at birth due to a higher infancy and childhood death rate in Nepal, we performed a sensitivity analysis by taking 70-year and 80-year life expectancies. The predicted HIV prevalence increased by 0.03% and 0.05% for 70-year and 80-year life expectancies, respectively. This shows that the predicted prevalence is less sensitive to *μ*, and we present results with *μ *= 0.022 y^-1^.

The estimated mean duration of infection without treatment varies widely from 8.6 years to 19 years [17,19,32,33]. The HIV/AIDS response in Nepal has largely focused on prevention and awareness, with less attention to the treatment, care, and support of persons living with HIV/AIDS [2]. For example, by the end of 2003, estimated 4,000 adults in Nepal needed antiretroviral therapy, but only 77 received the treatment as of June 2004. Since the health care system is poor and the education level is low, the life expectancy of HIV infected people is relatively short. Therefore, we assumed that the mean infection period is 8.6 years, which gives 1/*δ *= 8.6 years, i.e. *δ *= 0.116 y^-1^.

We fitted both the close form solution and the full SIM model to the HIV prevalence data and estimated parameters *λ*, *α*, *p*, *β *and *Λ *(See Appendix for a detailed description of data fitting and calculation of 95% confidence intervals).

## Results

### Model Validation

Estimated and all other parameters are given in Table 2. The HIV prevalence curve predicted by our model for Far-Western Nepal is in good agreement with the data (Figure 2).

**Table 2:** Model parameters for Far-Western Nepal.

Description	Parameter	Base Value [Low-High]	Source
Demographic Parameters			

Total sexually active population	*N *(0)	638,130	Estimated, Calculated, [28]
			
Initial compartments			
*Susceptible*	*S *(0)	419,610	Calculated
*HIV infected*	*I *(0)	210	Calculated
*Migrants in India*	*M *(0)	218,310	Estimated, Calculated, [11,12]
Maturation	*Λ*	15,321 (AS^a^)	Data fitting
		13,999 (FM^b^)	Data fitting
Death rate (non-HIV)	*μ*	0.022	Calculated, [31]
Migration rate			
*To India*	*α*	0.39 (AS)	Data fitting
		0.38 (FM)	Data fitting
*From India*	*λ*	0.024 (AS)	Data fitting
		0.028 (FM)	Data fitting
HIV^+ ^probability in returned migrants	*p*	0.022 (AS)	Data fitting
		0.020 (FM)	Data fitting

	HIV Disease Parameters		

Transmission rate	*β*	0.016 (AS)	Data fitting
		0.018 (FM)	Data fitting
HIV/AIDS death rate	*δ*	0.116 [0.116 - 0.053]	Estimated, Calculated, [2,17,19,32,33]

	Sexual Behavior Parameters		

Annual no. of sex partners	*n*	1.5 [1-5]	Estimated, [7]
Condom use	*c*	0.39 [0.12-0.60]	Estimated, Calculated, [6,7,9]
			
HIV transmission probability	*θ*	0.02 [0.01-0.11]	Estimated, [17,33,34]

^a ^AS: Analytic solution in low-prevalence setting; ^b ^FM: Full SIM model

We estimated the HIV prevalence among the returning migrants as 2.2% (95% CI: 2.0-2.3), consistent with a finding of 2.8% prevalence among the Mid-Far Western migrants returning from Mumbai, India [6]. We note that there are survey reports which indicate higher HIV prevalence among some returning migrants [7-9]. However, those studies were focused on the specific areas of Doti and Achham districts, and so do not represent overall Far-Western Nepal.

According to the World Factbook [31], the birth rate of Nepal is 30.46 births/1,000 population and the infant mortality rate is 63.66 deaths/1,000 live births. These provide the maturation rate of 2.85% of the population, which is in accordance with our estimate of *Λ *= 15,321 (95% CI: 15,303-15,339), i.e. 2.40% of the total population.

Among non-migrants surveyed by Poudel et al. [7], 75% did not have multiple sex partners in the last 5 years. Taking one sex partner for 75% of the population and on average 3 sex partners for the remaining 25% of the population, we get the average number of sex partners to be *n *= 1.5. Some studies show that the rate of condom use in Far-Western Nepal varies from 12% by married migrants with their wives [9] and 44% by non-migrants [7] in pre- or extra-marital sex to 60% by some migrants with sex workers [6]. Taking an average, the rate of condom use is 38.7% i.e. *c*~0.39. Using this value in our estimate of *β *= 0.02 (95% CI: 0.01-0.03), we obtained the transmission probability *θ *= 0.02, which is within the limit of 0.01-0.06 given in [17,33,34].

We obtained the rate of mobility to India as *α *= 0.39 (95% CI: 0.36-0.41) and the returning rate as *λ *= 0.024 (95% CI: 0.020-0.028). In the early 90s the seasonal migration to India was relatively low, as a 1994 survey conducted in 11 districts of the Far-Western and the Mid-Western regions found that 15% of adults migrated seasonally to India [35]. However, a decade of violent conflict (Maoist insurgency 1996-2006) dramatically exacerbated migration and displacement [36,37]. As a consequence, a recent estimate suggests an out-migration of up to 90% of adult men in some villages of the Far-Western hills [24,38]. The high mobility to India is also in agreement with the fact that one record in late December 2004 puts the figure at 200 Nepalis crossing the border every hour [39].

### Disease Outcomes: Prevalence and Infection

By the year 2015, the prevalence will still remain low (0.85%) among the general population (Table 3). The total new infections generated by the year 2015 due to the sexual activities back home is only 239 which indicates that the sexual activity back home is comparatively less vulnerable. However, by the year 2015, the number of Far-Western migrants living with HIV/AIDS in India will reach about 12,000 compared to 4,800 in 1990. Moreover, 7,000 HIV/AIDS will have been recruited from India, and the total deaths due to AIDS will reach about 36,000 by the year 2015.

**Table 3:** Effect of two prevention programs - PTG and PTM - on disease outcomes.

	A	B	C	D	E
Base case (39% condom use and *p *= 2.2%)	0.84	239	35,669	12,486	7,065
					
PTG^1^					
80% condom use	0.82	77	35,636	12,486	7,065
					
PTM^2 ^					
*p *= 1.1%	0.42	124	17,906	6,261	3,542
*p *= 0.5%	0.19	61	8,178	2,851	1,612

A: HIV Prevalence (%) among the general population, B: New Infection due to sexual activities back home, C: Disease Death, D: HIV^+ ^migrants in India, E: Total HIV infected individuals recruited from India, ^1^Prevention program targeted at the general population, ^2^Prevention program targeted at the migrants

### Effect of Seasonal Labor Migration on Disease Outcomes

**Figure 3:**
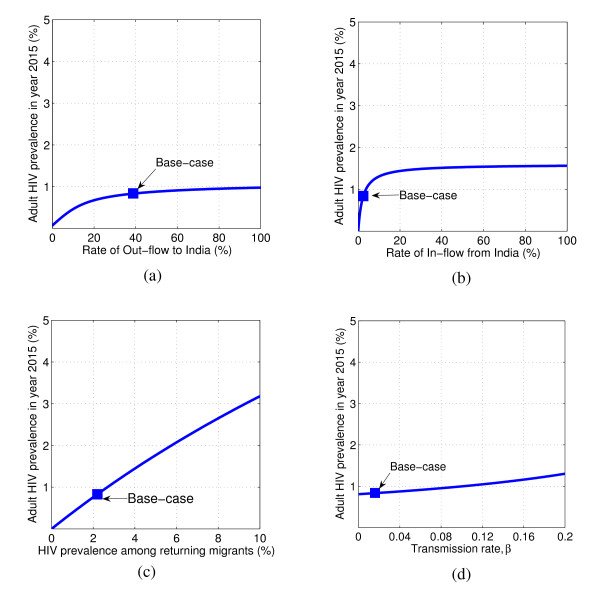
**HIV prevalence in the year 2015 in Far-Western Nepal**. Variation with respect to (a) rate of out-flow to India, *α*; (b) rate of in-flow from India, λ; (c) prevalence of HIV among returning migrants, *p*; and (d) transmission rate, *β*. Base-case indicates the HIV prevalence in the year 2015 predicted by the model using parameters in Table 2.

The migration has a negative impact on the disease outcomes as demonstrated by the HIV prevalence reached by the year 2015 among the general population in Far-Western Nepal for a varying rate of out-flow to India (Figure 3a), in-flow from India (Figure 3b) and HIV prevalence among the labor migrants in India (Figure 3c).

If the out-flow migration to India increases from the current rate of 39% to 60%, HIV prevalence in 2015 increases to 0.91%. In this case, 13,000 HIV infected migrants will be living in India in the year 2015 with the total recruitment of 7,500 HIV infections into Far-Western Nepal. Similarly, an increase in the rate of in-flow migration from India to 5% causes an increase of HIV prevalence to 1.11% with about 14,000 HIV infections entering Far-Western Nepal and 12,000 migrants living with HIV/AIDS in India in the year 2015.

HIV prevalence in the year 2015 among the general population in Far-Western Nepal increases linearly with a rate 0.32% per % increase in *p*, which represents the unsafe sexual activity level of migrants in their work place in India (Figure 3c). If the prevalence among the returning migrants from India reaches as high as 10% obtained in a 2001 survey conducted in some villages of Doti district [7,8], the prevalence among the general population will reach about 3.5% in the year 2015. This will project into a scary figure of 56,000 Far-Western migrants living with HIV/AIDS in their work place in India with a recruitment of 32,000 HIV infections to Far-Western Nepal and 159,000 deaths due to AIDS by the year 2015.

### Effect of Back Home Sexual Activity on Disease Outcomes

The sexual activity back home in the Far-Western region can be evaluated with the parameter *β*. In the absence of HIV infection due to migration, *β *also defines one of the important disease dynamic properties, the basic reproductive number, *R_0_*= *β*/(*α+ μ+δ*) [40,41] (See Appendix), which represents the effective number of secondary infections caused by a typical infected individual during his/her entire period of infectiousness [42]. The current basic reproductive number in Far-Western Nepal is *R_0_*= 0.03 < 1 indicating that the disease will theoretically be under control provided that the recruitment of HIV infection via the returning migrants is completely stopped. A change of sexual behavior resulting in an increase of *β *by 10-fold i.e. *β *= 0.18 leads to an increase of HIV prevalence to 1.22% in the year 2015 with an increase of the total new infections to 3,200 and the total death due to AIDS to 36,000 (Figure 3d).

### Effect of Prevention Programs on Disease Outcomes

We evaluated two illustrative prevention programs: one targeted at the general population (PTG) and another at the migrants (PTM). While PTG is focused on the behavioral interventions in the Far-Western region such as educating villagers to increase condom use and/or reduce the number of sexual partners, PTM is focused on the behavioral interventions in India such as reducing the frequency of brothel visits by migrants, particularly after drinking alcohol, or increasing safe sex practices in India.

In our model, varying condom use is similar to varying the number of sexual partners. If the average condom use increases from the current 39% to 80%, HIV prevalence in the year 2015 can be reduced by 0.02% to the prevalence of 0.82%, which decreases the total new infections due to the sexual activities back home from 239 to 77. Despite a significant reduction of new infections due to the sexual activities back home, PTG remains ineffective in controlling the HIV epidemics. More importantly, PTG does not help suppress the HIV infections due to the recruitment via returning migrants from India. About 12,000 migrants will still be living with HIV/AIDS, with the recruitment of 7,000 HIV infections from India (Table 3).

PTM, on the other hand, is significantly effective in reducing HIV prevalence both among the migrants and the general population. By the effective intervention that reduces *p *from 2.2% to 1.1%, HIV prevalence among the general population can be reduced to 0.42% as compared to 0.84% of the base case. With *p *= 1.1% only 6,300 migrants will be living with HIV/AIDS in India in the year 2015. Moreover, the number of HIV infections recruited via the returning migrants, the total death due to AIDS and the total new infections due to the sexual activities back home reduce to 3600, 800 and 130, respectively (Table 3). By reducing *p *further, the disease epidemic can significantly be mitigated (See Table 3 and Figure 3c).

## Discussion

Among the four major HIV epidemic regions of Nepal, i.e., highway districts (49% of HIV infections), Kathmandu valley (16%), Far-Western region (16%) and remaining hills (19%), the Far-Western region is unique in terms of the recruitment of HIV infections via seasonally returning migrants from India. Here we developed a mathematical model to evaluate the effects of seasonal labor migration to India on exacerbating the HIV burden in the Far-Western region of Nepal. Using the calibrated model, we assessed two prevention strategies - one focusing on back home and another on migrants in their work place in India.

Based on the USAID/WHO data for the whole country of Nepal, we derived HIV prevalence in the Far-Western region. By fitting our model to this derived data, we estimated the mobility rate of seasonal migrants to and from India, the rate of HIV prevalence among migrants, the transmission rate due to sexual activities back home and the maturation rate of the population. We calculated the number of HIV infected Far-Western migrants living in India, new infection due to back home sexual activities, HIV infections recruited from India via returning migrants and deaths due to AIDS by the year 2015.

The estimation from data fitting using our model predicts a high mobility rate of seasonal labor migrants from Far-Western Nepal to India. This high mobility is related to the most economic deteriorated condition of the Far-Western region and 10-year long violent conflicts (Maoist Insurgency 1996-2006) within the country. The Far-Western region has been the most affected region by this economic hardship, leading directly to a huge displacement of adult population to India [24,36-39], particularly to Mumbai. There is evidence of unsafe sexual activities of these migrants while they are working in India - for example, frequent brothel visits, mostly after drinking alcohol [7,8]. Such activities put these migrants at a high risk of contracting HIV and subsequently transmitting it to the Nepalese population upon their return. Our model predicts the HIV prevalence among these returning migrants to be 2.2%, which is consistent with a previous study [6]. However, this rate could be as high as 10% if the study is focused on some specific communities such as some Doti villages and Achham villages of the Far-Western region, where the vulnerability of HIV infections due to labor migrants is quite high [7-9]. The HIV prevalence among returning migrants can significantly exacerbate HIV prevalence among the general population (Figure 3c).

Compared to the total number of HIV infections recruited from India via seasonally returned migrants, the total number of new infections generated due to back home sexual activities is significantly low. This can also be reflected by the low reproductive number of 0.03 in the absence of the recruitment of HIV infections from India. This explains the current condition of low HIV prevalence of Nepal among the general population (less than 0.5% [4,5]) even though the high HIV prevalence among high-risk groups such as male migrants is one of the biggest concerns of the country's public health.

Our results show that HIV cannot be eradicated from the Far-Western region of Nepal unless the recruitment of HIV infections from India is stopped. Since the major source of HIV infections is the returning migrants rather than back home sexual activities, the prevention programs that educate villagers to promote safe-sex practices such as condom use are ineffective in suppressing the HIV burden in the region. Moreover, given the high mobility across the open-borders between Nepal and India, implementing a screening program at the borders is an infeasible immediate policy. Our model suggests that prevention programs focused on migrants to promote safe-sex activities in their workplace will be most effective on suppressing the HIV epidemic in Far-Western Nepal. In a survey study by Poudel et al. [20], the practice of safer sex in Mumbai by migrant workers has been reported after they received a package containing a letter with pamphlets about HIV/AIDS and condom packets. Such prevention programs that reduce the HIV prevalence among migrants can significantly lower the HIV prevalence among the migrants as well as the general population, new infections and disease deaths (Table 3). Therefore, programs that reduce the prevalence of HIV among migrants are urgently needed for a successful control of the HIV epidemic in Far-Western Nepal.

It is worth mentioning that population mobility to and from a region has been a fundamental characteristic of today's globalized world. The effect of such mobility on increasing the HIV burden has been evidenced in many parts of the world such as South Africa [43-45] and Australia [46]. Therefore, our model and analysis with necessary modifications might be applied to evaluate the HIV dynamics in other parts of the world where migration is playing a role in HIV epidemics.

## Conclusion

Mathematical models developed based on the disease characteristic in a particular region help better understanding the disease dynamics of that region and guide policy-makers in allocating resources for prevention and control of the infectious disease epidemic. We developed a mathematical model to explain the dynamic of HIV epidemics in Far-Western Nepal. Our SIM model suggests that the seasonal labor migration to India and their sexual activities in their workplace are the key factors contributing to the dynamics of the HIV epidemic in Far-Western Nepal. Any prevention strategy focused on activities of labor migrants in their workplace could provide an effective way of fighting against the HIV epidemic in Far-Western Nepal.

## Appendix

### Stable Equilibrium Prevalence

Let E* = (X*, M*, I*) denote a constant solution (steady state) of the model (4-6). Then using (*dM*/*dt*)|_(X*, M*, I*) _= 0, we get(7)

Using this in (*dI*/*dt*)| _(X*, M*, I*) _= 0, we get(8)

where(9)

For *p *= 0 (i.e. in the absence of the recruitment of HIV infection from India via seasonally returned migrants), *R*_0 _represents the basic reproductive number, which is defined as the effective number of secondary infections caused by a typical infected individual during his/her entire period of infectiousness.

Solving the quadratic equation (8), we obtain the following equilibrium prevalence:(10)

We note that  This implies that the equilibrium prevalence  increases as *p *increases. Moreover,  as *p→*∞. So, the maximum prevalence exists at the upper boundary of *p*. Therefore, the maximum prevalence can be reached at *p *= 1 and the maximum prevalence  is(11)

Solving (7), (10) and (*dX*/*dt*)|_(X*, M*, I*) _= 0, we obtain the following equilibrium:(12)(13)(14)

### Low-prevalence Scenario

In the low prevalence setting, we have(15)

Furthermore, to simplify the calculation, we assume that the total population remains constant so *N*(*t*) = *N_c_*. This requires

and hence(16)

Using (15) and (16), the model for the low prevalence scenario is(17)(18)(19)

The system of linear equations (17) - (19), with initial conditions *X*(0)=*X*_0_, *I*(0)=*I*_0_, *M*(0)=*M*_0_, can be solved in close form:(20)(21)(22)

### Data Fitting and Confidence Interval

We fit the solution (20) - (22) to the HIV prevalence data in Table 1 and estimate parameters *λ*, *α*, *p *and *β *by solving the following optimization problem:(23)

where(24)

Here,  is the HIV prevalence data at time point *t_i_*given in Table 1. We used *t*_1990 _= 0, the initial time for the computation. The parameter *Λ *can be approximated by using the mean-value,  where *t_f_*is the final time-point considered.

Furthermore, we estimated the parameters *λ*, *α*, *p*, *β *and *Λ *by fitting the full SIM model (1) - (3) to the data (Table 1). Since the estimated parameters (Table 1) and the predicted curves (Figure 2) by both SIM model (1) - (3) and the solution (20) - (22) are almost the same, the expressions (20) - (22) provide a reasonably good approximation to the solution of the SIM model.

**Figure 4:**
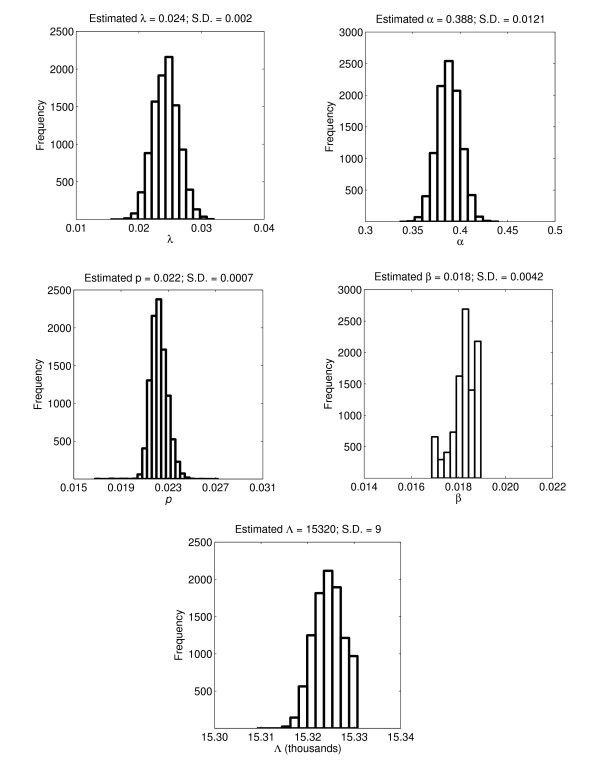
**The frequency distribution from bootstrap method**. The distribution of the parameter estimates obtained from 10,000 simulations using parametric bootstrap method.

For the least-squares fitting procedure, we used the built-in routine *fminsearch.m *in the optimization toolbox of MATLAB (The Mathworks, Inc.), which implements the simplex search method. We also obtained the standard deviation of the parameters from a simulation study of 10,000 bootstrap replicates. The frequency distribution is presented in Figure 4.

## Competing interests

The authors declare that they have no competing interests.

## Authors' contributions

NKV conceived and organized the study; performed the model analysis, data fitting and simulations; and wrote the manuscript. JW contributed in conceiving and designing the study, and writing the manuscript. Both authors read and approved the final manuscript.

## Pre-publication history

The pre-publication history for this paper can be accessed here:

http://www.biomedcentral.com/1471-2458/11/310/prepub
